# Automated Restarting Fast Proximal Gradient Descent Method for Single-View Cone-Beam X-ray Luminescence Computed Tomography Based on Depth Compensation

**DOI:** 10.3390/bioengineering11020123

**Published:** 2024-01-26

**Authors:** Peng Gao, Huangsheng Pu, Tianshuai Liu, Yilin Cao, Wangyang Li, Shien Huang, Ruijing Li, Hongbing Lu, Junyan Rong

**Affiliations:** 1 School of Biomedical Engineering, Air Force Medical University, Xi’an 710032, China; xiangzhiwanli@163.com (P.G.); liutianshuai91@126.com (T.L.); easylinmonk@163.com (Y.C.); liwangyang1995@163.com (W.L.); 18637881307@163.com (S.H.); liruijing1025@163.com (R.L.); 2 College of Advanced Interdisciplinary Studies & Hunan Provincial Key Laboratory of Novel NanoOptoelectronic Information Materials and Devices, National University of Defense Technology, Changsha 410073, China; rookiego@yeah.net; 3 Nanhu Laser Laboratory, National University of Defense Technology, Changsha 410073, China

**Keywords:** image reconstruction, X-ray luminescence computed tomography, optical tomography, ill-posed

## Abstract

Single-view cone-beam X-ray luminescence computed tomography (CB-XLCT) has recently gained attention as a highly promising imaging technique that allows for the efficient and rapid three-dimensional visualization of nanophosphor (NP) distributions in small animals. However, the reconstruction performance is hindered by the ill-posed nature of the inverse problem and the effects of depth variation as only a single view is acquired. To tackle this issue, we present a methodology that integrates an automated restarting strategy with depth compensation to achieve reconstruction. The present study employs a fast proximal gradient descent (FPGD) method, incorporating L0 norm regularization, to achieve efficient reconstruction with accelerated convergence. The proposed approach offers the benefit of retrieving neighboring multitarget distributions without the need for CT priors. Additionally, the automated restarting strategy ensures reliable reconstructions without the need for manual intervention. Numerical simulations and physical phantom experiments were conducted using a custom CB-XLCT system to demonstrate the accuracy of the proposed method in resolving adjacent NPs. The results showed that this method had the lowest relative error compared to other few-view techniques. This study signifies a significant progression in the development of practical single-view CB-XLCT for high-resolution 3−D biomedical imaging.

## 1. Introduction

X-ray luminescence computed tomography (XLCT) is a novel hybrid imaging technique that combines X-ray radiography and optical luminescence tomography in a synergistic manner [1,2,3]. In XLCT, the process involves the utilization of targeted X-ray irradiation to stimulate specific nanophosphors (NPs) that are introduced into the tissues of interest. The luminescent signals produced as a result are then detected and used to reconstruct three-dimensional (3−D) images. These images offer supplementary anatomical, functional, and molecular information. Compared to computed tomography (CT) and magnetic resonance imaging (MRI), XLCT provides enhanced functional and molecular imaging capabilities. This is achieved through the utilization of targeted NPs that enable the visualization of molecular processes, such as gene expression and enzyme activity. Furthermore, the utilization of X-rays as the excitation source in XLCT offers enhanced depth penetration and resolution for soft tissue imaging, surpassing the capabilities of pure optical techniques, such as fluorescence molecular tomography (FMT) [4,5] and bioluminescence tomography (BLT) [6,7]. XLCT’s distinctive capability to generate 3−D images with exceptional sensitivity, resolution, and penetration depth positions it as a promising and emerging tool for multifunctional imaging.

First introduced in 2010 by Pratx et al. [1], XLCT has experienced substantial advancements over the past decade, encompassing the development of NPs, imaging systems, and reconstruction techniques. Various types of NPs have been the subject of extensive research [8]. Notably, NPs such as LaAlO_3_:Eu^3+^ [9] and NaGdF_4_:Eu^3+^ [10] have demonstrated exceptional performance in terms of X-ray excitation efficiency, luminous efficiency, and chemical stability. Advanced XLCT systems, such as pencil/narrow-beam XLCT (PB-XLCT or NB-XLCT) [1,11,12,13,14] and cone-beam XLCT (CB-XLCT) [2,15,16,17], have been proposed. PB-XLCT offers superior image quality through selective excitation, albeit at the cost of longer scan durations. While certain techniques, such as the continuous scanning scheme [18,19], have been shown to enhance imaging efficiency, their imaging geometry necessitates linear steps that are not conducive to rapid imaging, such as in the case of drug biokinetics [20]. The scanning time of cone-beam XLCT is significantly reduced due to the simultaneous irradiation of the entire object. Nevertheless, the spatial resolution of the imaging is compromised due to the ill-posed nature of the reconstruction process. Additionally, the size and location of the X-ray beam cannot be utilized as prior information during the reconstruction of PB-XLCT.

To enhance the imaging performance of CB-XLCT, researchers have made significant contributions from various perspectives. Model-based iterative reconstruction algorithms, such as the Bayesian framework-based method proposed by Zhang et al. [16], have the capability to automatically estimate regularization parameters and effectively preserve target edges. To enhance the quality of images obtained from a reduced number of projections, which is advantageous for rapid biomedical imaging applications, the utilization of compressed sensing (CS) theory has been employed for reconstruction. Various methods, including the L1-TV method [21], robust elastic net-ℓ1ℓ2 method [22], and T-FISTA method [23], have been utilized to achieve this goal. With the utilization of sparsity or group sparsity as a prior, our research team has successfully shown that CB-XLCT has the capability to differentiate between two targets with an edge-to-edge distance (EED) of 0.1 cm by employing two imaging views [24]. Although the acquisition time is notably decreased by using two views, it is important to note that the imaging object undergoes inevitable rotation during the view span, which is deemed unacceptable in numerous clinical scenarios. Thus, the proposal of single-view CB-XLCT has been put forward [15,25,26]. Current single-view CB-XLCT reconstruction demonstrates satisfactory performance in scenarios where there is a single target or multiple targets with prior CT information [27]. Challenges persist in the reconstruction of single-view multitarget CB-XLCT.

Single-view CB-XLCT imaging presents a complex ill-posed inverse problem, particularly when targets are situated at different depths, resulting in nonlinear depth sensitivity [28]. This paper employs two distinct strategies in order to address the challenge of single-view reconstruction. Firstly, in order to address the issue of depth inconsistency in single-view CB-XLCT, the technique of depth compensation is implemented to enhance the quality of imaging. Secondly, in order to address the ill-posed nature of the inverse problem, an automated restarting strategy is implemented during the reconstruction process. Given the satisfactory performance of L0-norm-based CB-XLCT reconstruction in single-view imaging, this study employs a fast proximal gradient descent (FPGD) method [29] to facilitate L0-norm-regularized sparse reconstruction, thereby enhancing convergence speed. This method exhibits three significant advantages in comparison to prior research. Firstly, it has the capability to recover adjacent multitarget distributions without the need for CT priors, as demonstrated in Liu’s work [27]. This showcases its wide applicability beyond fluorescence and bioluminescence tomography. Secondly, the implementation of the automated restarting strategy ensures the generation of reliable reconstructions without the need for manual intervention, as opposed to our previously proposed re-pdNCG method [28]. Thirdly, imaging performance is only marginally influenced by the positioning of the target and detector within a specific range.

To assess the effectiveness of the proposed methodology, numerical simulations and physical phantom experiments were conducted using a custom-made CB-XLCT system. In the context of the numerical simulations, a three-dimensional (3−D) digital mouse was employed as the imaging object, with luminescent targets representing tumors embedded within it. In the conducted physical phantom experiments, a cylinder phantom was utilized, wherein two transparent tubes containing NPs (Y_2_O_3_:Eu^3+^) were immersed. Two algorithms previously proposed by our research group, namely T-FISTA [23] and DC-FL [24], were utilized for the purpose of comparing the sparse-view and limited-view CB-XLCT imaging techniques. Our findings demonstrated that the accurate resolution of luminescent targets is achievable from a single-view image.

The subsequent sections of this paper are structured in the following manner. The forward and inverse problems of single-view CB-XLCT are discussed in Section 2 (Section 2.1 and Section 2.2). The numerical simulations and physical phantom experiments that were conducted to validate the reconstruction performance of the proposed algorithm are presented in Section 2 (Section 2.3). The results are summarized in Section 3. Section 4 presents the discussions and conclusions.

## 2. Materials and Methods

### 2.1. Photon Propagation Model of CB-XLCT

In CB-XLCT imaging, X-rays travel through imaging objects and can be converted into light by NPs. The X-ray intensity at position **r** and the generated light source density can be described as follows:

(1) $$X(r)=X(r_{0})\exp\{-\int_{r_{0}}^{r}\mu_{t}(\tau )d\tau \}$$

(2) S(r)=ηX(r)ρ(r)

where *X*(**r**_0_) is the X-ray intensity at the initial position **r**_0_, *μ_t_(τ)* is the reconstructed X-ray attenuation coefficient, *η* is the light yield, and *ρ*(**r**) is the nanophosphor concentration at position r. 

Due to the weakly absorbing and highly scattering properties of biological tissues in the visible and NIR spectral windows, light transportation in tissues can be modeled by a diffusion equation (DE) with Robin-type boundary conditions [6]:

(3) $$\left\{\begin{array}{l} -\nabla [D(r)\nabla \Phi (r)]+\mu_{a}(r)\Phi (r)=X(r)\;(r\in \Omega )\\ \Phi (r)+2\kappa D(r)[\nu \nabla \Phi (r)]=0\;\;\;\;\;\;(r\in \partial \Omega ) \end{array}\right.$$

where *D*(**r**) is the diffusion coefficient (which can be calculated by *D*(**r**) = (3(μ_a_(**r**) + $\mu_{s}^{’}$(**r**)))^−1^, where μ_a_ and $\mu_{s}^{’}$ are the absorption and reduced scattering coefficients of the tissue, respectively), Φ(**r**) is the photon fluence at position r, Ω is the domain of the imaging object, ∂Ω denotes the boundary of Ω, κ is a constant describing the optical reflective index mismatch, and ν is the outward unit normal.

Based on the finite-element method (FEM), Equation (3) can be further discretized into the following matrix equation:

(4) FηAρ=KΦ

with

(5) $$\left\{\begin{array}{l} F_{ij}=\int_{\Omega }\Phi (r)\psi_{i}\psi_{j}dr\\ A_{ij}=A_{ij}(r)\\ K_{ij}=\int_{\Omega }(D\nabla \psi_{i}\nabla \psi_{j}+\mu_{a}\psi_{i}\psi_{j})dr+\frac{1}{2\alpha }\int_{\partial \Omega }\psi_{i}\psi_{j}dr \end{array}\right.$$

where *F_ij_*, *K_ij_*, and *A_ij_* are the elements of matrices **F***,* **K**, and **A**, respectively, and *ψ_i_* and *ψ_j_* are the corresponding elements of test function.

Equation (5) can be converted into a linear relationship between the nanophosphor concentration *ρ* and photon measurements on the object surface **Φ**_meas_.

(6) $$H\rho =\Phi_{\mathrm{meas}}$$

where **H** is a weight matrix with **H** = **K^−^**^1^**F***η***A**.

### 2.2. Automated Restarting CB-XLCT Reconstruction with Depth Compensation

In CB-XLCT, NPs are sparsely distributed in most biological applications and the luminescent targets are usually small compared to the entire reconstruction regions, especially when nanophosphors accumulate in early tumors. Thus, the compressive sensing (CS) technique can be adopted and Equation (6) can be solved with an L0-norm regularization to obtain sparse results as follows:

(7) $$\mathrm{minimize}\;f(\rho )={\left\|H\rho -\Phi_{\mathrm{meas}}\right\|}^{2}+\lambda {\left\|\rho \right\|}_{0}$$

where λ is the regularization parameter. 

It is known that detection sensitivity in optical imaging decreases nonlinearly as depth increases [30]. This causes CB-XLCT measurements to become hypersensitive to targets near the detector, a common occurrence in the single-view imaging of multiple targets. The resulting ill-posed weight matrices lead to reconstruction biases favoring superficial targets. Therefore, additional information should be provided to correct this nonlinear depth sensitivity.

To obtain effective depth compensation, a data weight matrix **W**_d_ and model weight matrix **W**_m_ are introduced in Equation (7).

(8) $$\left\{\begin{array}{l} \mathrm{minimize}\;f(\chi )={\left\|HW_{d}W_{m}^{-1}\chi -\Phi_{\mathrm{meas}}\right\|}^{2}+\lambda {\left\|\chi \right\|}_{0}\\ \chi =W_{m}\rho \end{array}\right.$$

**W**_d_ provides a weighting of or a constraint onto the solution, in which some a priori information, such as internal structural knowledge from CT or MRI modality or an initial estimate of the unknown parameter distribution, can be incorporated. **W**_d_ can be constructed as follows:

(9) $$W_{d}=\mathrm{diag}(\tilde{N})=\left(\begin{array}{ccc} \tilde{n_{1}^{q}} & \cdots & 0\\ \vdots & \ddots & \vdots \\ 0 & \cdots & \tilde{n_{n}^{q}} \end{array}\right)$$

where $\tilde{N}$ is a rough approximation of the true solution **N** and *q* controls the compromise between a close fit to the data and the stability of the solution. In this paper, the standard Tikhonov regularization reconstruction result was adopted as a priori information, considering the robustness of the imaging performance. The value of *q* was empirically set from 0.4 to 0.8, according to the reconstruction results.

**W**_m_ is used to level differences in detection sensitivities and can be given by

(10) $$\left\{\begin{array}{l} \beta_{j}=\frac{1}{\left|\max(A_{j})-\min(A_{j})\right|}\\ W_{m}=\mathrm{diag}\left\{\beta_{j}*||A_{j}|{|}_{2}^{-1}\right\}=\left(\begin{array}{ccc} \beta_{j}*||A_{j}|{|}_{2}^{-1} & \cdots & 0\\ \vdots & \ddots & \vdots \\ 0 & \cdots & \beta_{n}*||A_{n}|{|}_{2}^{-1} \end{array}\right),\;j=1,2,\cdots ,N \end{array}\right.$$

where *β_j_* is a normalization factor that is inversely proportional to the absolute largest difference between the elements within each column and *A_j_* is the *j*-th column of the matrix *A*. **W**_m_ is composed of two parts: the first part *β_j_* is used to compensate for differences in the relative sizes of elements within a column of the matrix *A* and the second part *A_j_* is used to compensate each element in a column with the average distance from the corresponding voxel to all of the source–detector pairs.

The L0-norm regularization problem is NP-hard in general and efficient methods, such as orthogonal least squares (OLS) [31] and orthogonal matching pursuit [32], have been proposed. Bao et al. [33] used a proximal gradient descent (PGD) method to find an approximate solution to the L0-norm regularization problem, which proved to have a sublinear convergence rate, with satisfactory empirical results. In this paper, we employed a fast proximal gradient descent (FPGD) method to solve Equation (8), which can be described as follows:

Initialization: The regularization parameter λ = 1e^−3^, the initialization $\chi_{1}=\chi_{0}$ = 1, t_0_ = 0;

Step k (k≥1): 

$u_{k}=\chi_{k}+\frac{t_{k-1}-1}{t_{k}}(\chi_{k}-\chi_{k-1})$

$z_{k}=P_{\sup(x_{k})}(u_{k})$

$\chi_{k+1}={\mathrm{prox}}_{sh}(z_{k}-s\nabla g(z_{k}))$

$t_{k+1}=\frac{1+\sqrt{1+4t_{k}^{2}}}{2}$

where P**_Q_**(**u**) indicates the novel support projection operator which returns a vector. The details of this process can be found in [29].

To alleviate the ill-posed nature of single-view CB-XLCT reconstruction, an automated restarting strategy was proposed with two-level iteration. In the inner iteration, the FPGD method was used to solve Equation (8) to obtain the $\chi_{k}$, values and the negative values of $\chi_{k}$ were set to zero. Then, a new optimization matrix was constructed by removing the columns in **H** corresponding to the zero values of $\chi_{k}$ as the outer iteration. The ill-posed nature could be alleviated by every outer iteration within a reset permissible region. The error threshold to stop the inner iteration was set to 1.0 × 10^−5^ and the regularization parameter λ was set to 1e-1, according to [29]. The outer iteration was automatically stopped when the 95% values of $\chi_{k}$ were zero and the initial guess $\chi_{0}$ was set to 1. The regularization parameter λ was set to 1.0 × 10^−3^. These parameters were selected empirically by comparing the reconstruction results to the actual shapes and positions of the luminescent targets. 

**Figure 1:**
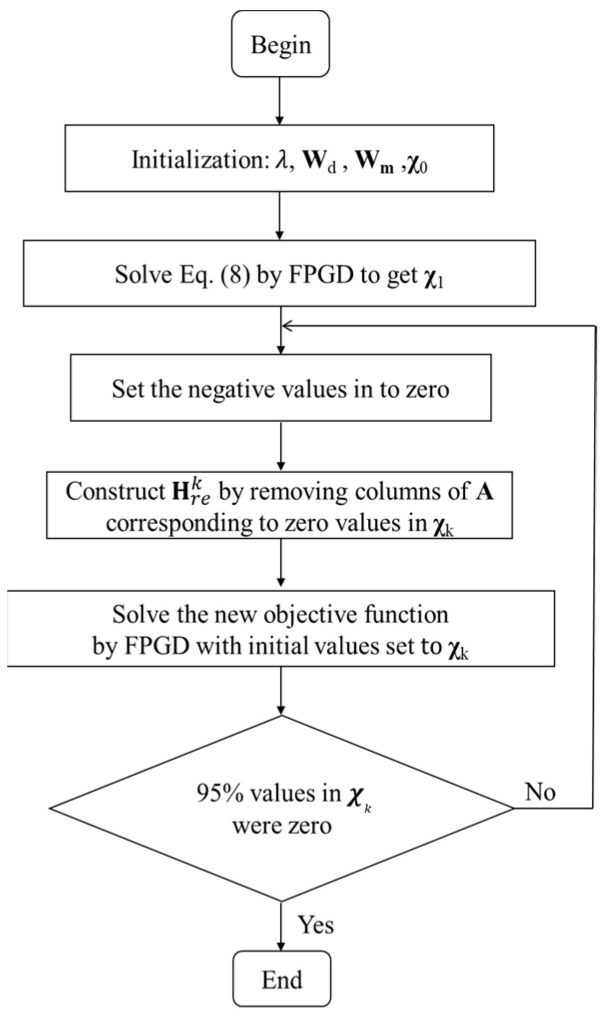
Flowchart of the re-DC-FPGD method.

The implementation of the proposed restarting FPGD method with depth compensation (re-DC-FPGD) is summarized in Figure 1.

### 2.3. Experimental Setup

#### 2.3.1. Numerical Simulations 

**Figure 2:**
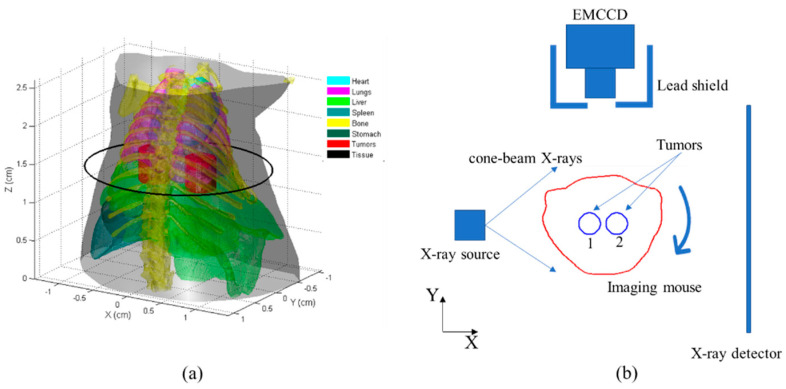
Schematic of the numerical simulations: (**a**) 3−D digital mouse with main organs and tumors (the investigated region was 2.6 cm in height), where the black circles depict the central slices of the tumors (represented in red); (**b**) CB-XLCT imaging system with initial phantom position setup, where the red line depicts the tomographic outline of the mouse and the two blue circles depict the tumors with NPs.

Numerical simulations were initially conducted using a digital mouse as the imaging object in order to evaluate the effectiveness of the proposed method. The height of the mouse was 2.6 cm and it contained the primary organs, including the heart, lungs, liver, spleen, bone, and stomach. Additionally, two cylindrical tumors, measuring 3 mm in height and 4 mm in diameter, were inserted into the liver of the mouse (Figure 2a). The scattering and absorption coefficients were allocated to the organs based on the study conducted by [34].

The configuration of the CB-XLCT system is depicted in Figure 2b. An electron-multiplying charge-coupled device (EMCCD) camera, positioned at a 90° angle relative to the X-ray axis, was employed to capture the luminescent images. An X-ray detector was employed to gather the transmitted X-ray signals. The mouse was positioned on a rotation stage, with the z-axis designated as the rotational axis and the Z = 0 cm plane defined as the bottom plane. The two tumors were positioned to be perpendicular to the X-ray detector at the initial position. The mouse was subjected to cone-beam X-rays with a tube voltage of 40 kV and a tube current of 1 mA. White Gaussian noise was introduced to the boundary measurements with a signal-to-noise ratio (SNR) of 20 dB. To assess the imaging performance of the re-DC-FPDG method, three simulation cases were performed. In each case, the two tumors were positioned at the varying edge-to-edge distances (EEDs) of 0.3 cm, 0.2 cm, and 0.1 cm.

#### 2.3.2. Physical Phantom Experiments 

Physical phantom experiments were conducted using the CB-XLCT system, which was custom-made by our laboratory. Reference [35] provides comprehensive information regarding the system’s configuration.

The physical phantom utilized in the experiment comprised a transparent glass cylinder with a diameter of 3.0 cm and a height of 7.0 cm. The cylinder was filled with a mixture of 1% intralipid and water. The absorption coefficient and the reduced scattering coefficient of the medium were μ_a_ = 0.02 cm^−1^ and 10 cm^−1^, respectively. Two glass tubes, each with a diameter of 0.4 cm, were utilized to house Y_2_O_3_:Eu^3+^ nanoparticles with a concentration of 0.1 g/mL. These tubes were then implanted into the phantom. Two experimental cases were conducted to investigate the effects of the EED of two tubes. In case 1, the EED was set to 0.50 cm, while in case 2, it was set to 0.23 cm. These experiments were conducted in a manner similar to simulations.

For the purpose of single-view CB-XLCT imaging, the X-ray tube voltage was set to 40 kV and the current was set to 1 mA. The electron-multiplying (EM) gain, integration time, and binning settings of the electron-multiplying charge-coupled device (EMCCD) were configured to 260, 500 ms, and 1 × 1, respectively. For the purpose of CT imaging, a total of 360 images were acquired at 1° intervals. The CT reconstruction utilized the Feldkamp–Davis–Kress (FDK) algorithm [36].

**Figure 3:**
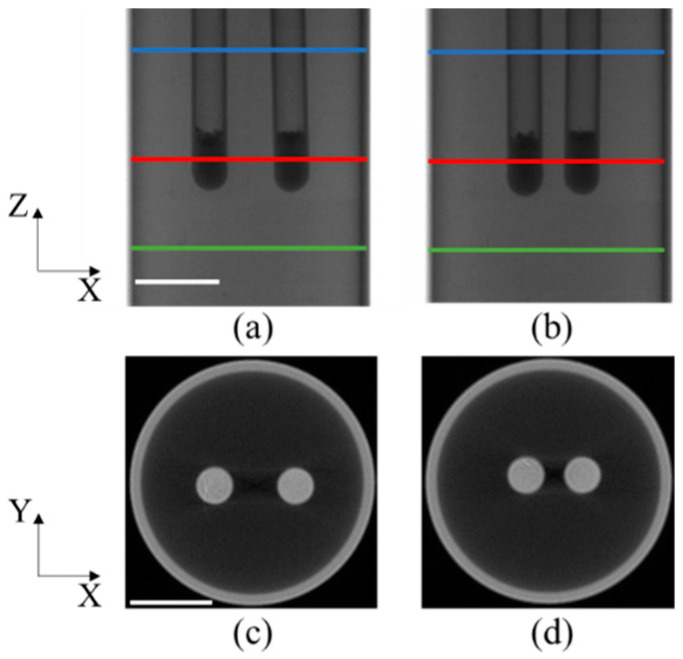
Setup of the phantom experiments: (**a**,**b**) representative X-ray projections of the phantom in case 1 and case 2. Regions between the blue and green lines are used for the study; (**c**,**d**) CT slices indicated by the red lines shown in (**a**,**b**).

Figure 3c,d displays the representative X-ray projections and tomographic images, respectively, of the phantom experiments for the initial position of the phantom. The area of interest under investigation was the region bounded by the green and red lines, measuring 2.6 cm in height. The tomographic images indicated by the blue lines in Figure 3a,b are represented as Figure 3c and Figure 3d, respectively. The tubes on the left and right sides are designated as tube 1 and tube 2, respectively.

#### 2.3.3. Quantitative Evaluation

The reconstructed images were compared using several indices, including location error (LE) [15], dice similarity coefficient (DICE) [37], and spatial resolution index (SPI) [23]. 

LE is defined as the Euclidean distance between the reconstructed and true positions of a target.

(11) $$\mathrm{LE}=||p_{r}-p_{t}|{|}_{2}$$

where p*_r_* denotes the maximum value position of the reconstructed target and p*_t_* denotes the real center position. 

DICE is used to evaluate the similarity between reconstructed and true luminescent areas to assess the quality of morphological reconstructions.

(12) $$\mathrm{DICE}=\frac{2|ROI_{r}\cap ROI_{t}|}{\left|ROI_{r}|+|ROI_{t}\right|}$$

where ROI*_r_* and ROI*_t_* denote the reconstructed and true luminescent areas, respectively. The higher the DICE value, the higher the similarity between the target in the reconstructed image and the original position.

SPI denotes the spatial resolutions of two targets and is defined as

(13) $$\mathrm{SPI}=\frac{\rho_{\max}^{l}-\rho_{\mathrm{valley}}^{l}}{\rho_{\max}^{l}-\rho_{\min}^{l}}$$

where *ρ^l^* denotes the value of the profile along a given line that connects the two centers of the reconstructed cross-sections and $\rho_{max}^{l}$, $\rho_{valley}^{l}$ and $\rho_{min}^{l}$ are the maximal, valley, and minimal values between the two peak values, respectively. Large SPI values indicate the high spatial resolutions of reconstructed images. 

In this paper, to quantitatively evaluate the performance of the proposed method, two CB-XLCT reconstruction methods previously proposed by our group were used for comparison, including the sparse-view-based T-FISTA method and the limited-view-based DC-FL method. The iteration numbers were chosen empirically according to the results and to ensure the convergence of the calculations. For XLCT reconstruction, each phantom was discretized into 2124 nodes and 9765 tetrahedral elements using COMSOL Multiphysics 3.3 (COMSOL, Inc., Burlington, MA, USA), which corresponded to normal discretization. It should be noted that with finer discretization, the computation time costs would increase substantially but the spatial resolution would not be improved since the optimization difficulty would increase accordingly. The proposed algorithm was implemented on an Intel 3.40-GHz processor and 32-GB RAM personal computer and the calculation time was about 14.2 s.

## 3. Results

### 3.1. Numerical Simulations

**Figure 4:**
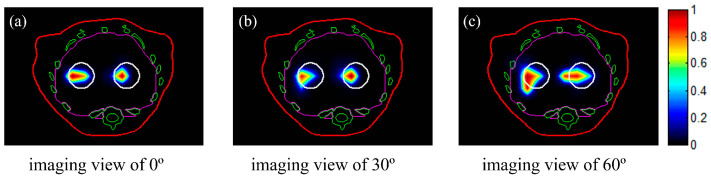
Reconstructed images of the tumors with NPs at different imaging views. The slices are those indicated with black circles in Figure 2. The white circles represent the real positions of the tumors. The red, green, and magenta lines represent the boundaries of the animal’s body, bones, and liver, respectively: (**a**–**c**) imaging views of 0°, 30°, 60°, respectively. All images were normalized to the maximal value.

To demonstrate the performance of the proposed method for different imaging views, reconstructions in case 1 were conducted at imaging views of 0°, 30°, and 60°, with an EED of 0.3 cm. The reconstructed 2D slices are shown in Figure 4. All of the slices depicted in Figure 4 were taken at a height of Z = 1.3 cm. The two tumors could be effectively identified using the three single views with the proposed method. In the following simulations, all reconstructions were conducted using the luminescent image at a viewing angle of 30º, taking into account differences in tumor depth.

**Figure 5:**
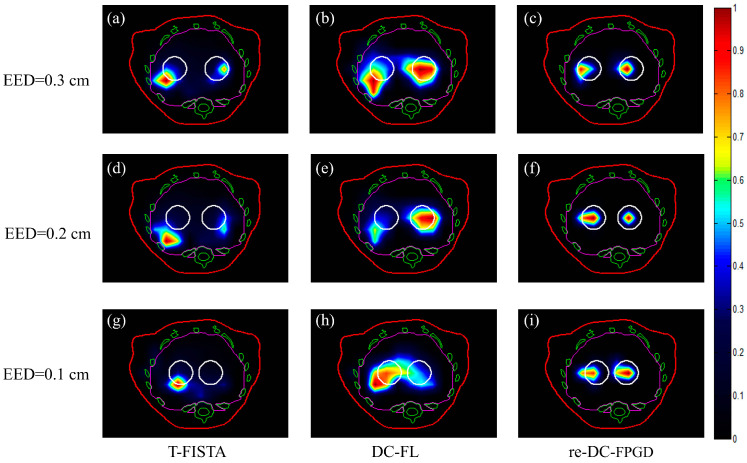
Reconstructed images of the tumors in different cases using the three methods. The white circles represent the real positions of the tumors. The red, green, and magenta lines represent the boundaries of the animal’s body, bones, and liver, respectively (**a**–**c**) tomographic slices reconstructed by T-FISTA, DC-FL, and the proposed re-DC-FPGD algorithm with an EED of 0.3 cm. (**d**–**f**) tomographic slices reconstructed by different algorithms with an EED of 0.2 cm. (**g**–**i**) tomographic slices reconstructed by different algorithms with an EED of 0.1 cm.

The reconstructed 2D slices of the three cases obtained by the T-FISTA, DC-FL, and proposed re-DC-FPGD algorithms are shown in Figure 5. The three columns in Figure 5 represent the reconstruction results obtained using the different methods, while the rows represent the different EEDs.

The results indicated that the sparse-view-based T-FISTA method and the limited-view-based DC-FL method could not resolve the two tumors in all cases, as shown in the first and second columns of Figure 5. This was due to either a lack of depth compensation or the highly ill-posed nature of the reconstruction problem. With the revised strategy that incorporates depth compensation, it was possible to clearly recover the two targets, even when they were in close proximity (EED = 0.1 cm), as depicted in Figure 5i.

**Figure 6:**
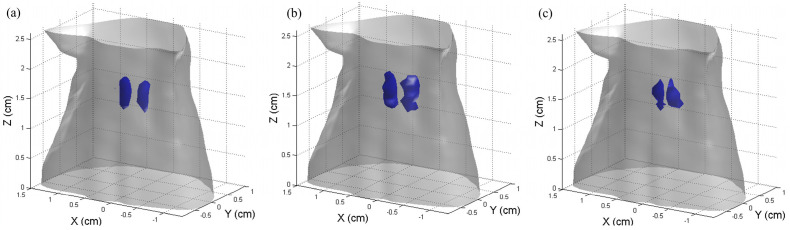
The 3−D results of the reconstructed CB-XLCT images using the proposed method in the simulations of the three cases: (**a**–**c**) case 1 to case 3. Tumors are represented in blue.

Figure 6 shows the 3−D results of the reconstructed CB-XLCT images using the proposed method. The blue objects represent the tumors. As expected, in all cases, the re-DC-FPGD method could clearly distinguish the two targets using a single-view luminescent image.

The quantitative analysis results of case 3, obtained using the different methods, are presented in Table 1. Among the three methods compared, the proposed re-DC-FPGD method achieved the lowest LE, the highest DICE, and the highest SPI. This suggests that the re-DC-FPGD method outperformed the others in terms of localization accuracy, shape recovery, and the preservation of spatial resolution when reconstructing the target.

### 3.2. Numerical Simulations

**Figure 7:**
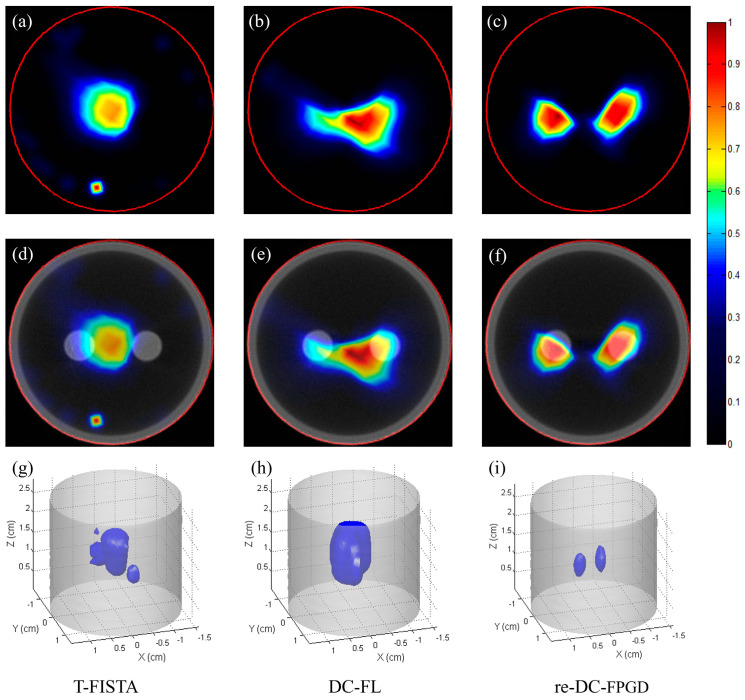
Phantom experiment results for case 1: (**a**–**c**) CB-XLCT slices reconstructed by T-FISTA, DC-FL, and the proposed re-DC-FPGD algorithm, respectively. (**d**–**f**) CB-XLCT/CT fusion results of different methods. (**g**–**i**) 3−D rendering results of different methods. The red circles in the CB-XLCT images depict the boundaries of the phantom. The blue objects in the 3−D renderings represent the recovered targets.

Figure 7 presents the CB-XLCT reconstruction results for case 1 of the phantom experiments, which had an EED of 0.50 cm. The reconstructed images were based on the luminescent images taken at a 30° viewing angle. All images were normalized to the maximum intensity value. The first row of Figure 7 shows the reconstructed images (indicated by the red line in Figure 3a) using the T-FISTA, DC-FL, and proposed re-DC-FPGD methods. The second row displays the fused CB-XLCT/CT images, while the third row shows the 3−D rendering results. As expected, both the sparse-view-based T-FISTA and the limited-view-based DC-FL methods failed to separate the two targets. More noise is evident in Figure 7a compared to Figure 7b,c, indicating that the DC-FL and proposed methods performed better in terms of noise suppression. Owing to the depth compensation and the automatic restarting strategy, the proposed method clearly resolved the two tubes with a high contrast-to-noise ratio (CNR). The 3−D results (Figure 7h–j) further demonstrate the superiority of the proposed method over the other two.

**Figure 8:**
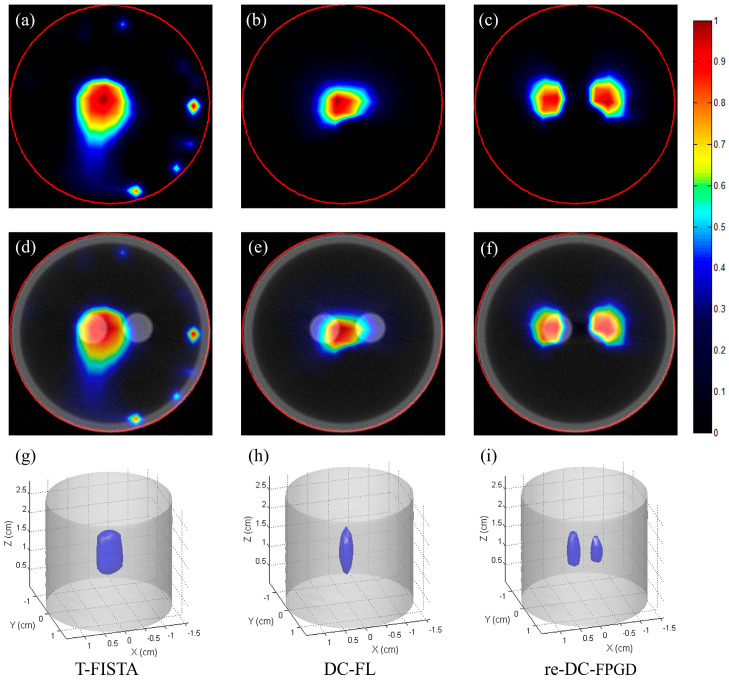
Phantom experiment results for case 2: (**a**–**c**) CB-XLCT slices reconstructed by T-FISTA, DC-FL, and the proposed re-DC-FPGD algorithm, respectively. (**d**–**f**) CB-XLCT/CT fusion results of different methods. (**g**–**i**) 3−D rendering results of different methods. The red circles in the CB-XLCT images depict the boundaries of the phantom. The blue objects in the 3−D renderings represent the recovered targets.

Figure 8 presents the single-view CB-XLCT reconstruction results for the phantom experiments for case 2, which had an EED of 0.23 cm. Figure 8a–c show the CB-XLCT slices (indicated by the red line in Figure 3b) obtained using the T-FISTA, DC-FL, and proposed re-DC-FPGD algorithms, respectively. Figure 8d–f display the corresponding XLCT/CT fusion results. Figure 8h–j show the results of the 3−D rendering. Similar to case 1, both the T-FISTA and DC-FL methods failed to achieve high image quality. In contrast, the re-DC-FPGD approach achieved superior spatial resolution, as demonstrated by the tomographic images (Figure 8c,f) and 3−D results (Figure 8j). The location accuracy and shape recovery achieved by the re-DC-FPGD reconstruction demonstrated its ability to produce high-quality single-view CB-XLCT images, even at the low EED of 0.23 cm.

Table 2 presents the results of the quantitative analysis of the reconstructions in the phantom experiments for case 2. In line with the numerical simulation findings, the proposed re-DC-FPGD method achieved the lowest LE, the highest DICE, and the highest SPI compared to the other two methods. This demonstrates that even for physical phantom experiments at the low EED of 0.23 cm, the re-DC-FPGD algorithm demonstrated superior performance in terms of target localization accuracy, shape recovery, and the preservation of structural details compared to T-FISTA and DC-FL. The quantitative metrics supported the observed improvements in visual quality in the re-DC-FPGD phantom reconstructions.

## 4. Discussion

Single-view CB-XLCT imaging enables the fast and noninvasive 3−D visualization of NP distributions during biological processes, making it highly attractive for biomedical research. However, the reconstruction of CB-XLCT from a single view poses a severely ill-posed inverse problem since only one view of the data is acquired. To address this, the present work utilized automated restarting FPGD combined with depth compensation (re-DC-FPGD) to tackle single-view CB-XLCT reconstruction. Together, these strategies enabled high-quality single-view CB-XLCT reconstruction, as evidenced by simulations and phantom studies.

The simulation and phantom experiment results demonstrated that the proposed re-DC-FPGD method could accurately resolve two adjacent targets, even at the low EED of 0.1 cm (Figure 5i). In contrast, the targets could not be distinguished using the T-FISTA or DC-FL approaches. Quantitative evaluation showed that the re-DC-FPGD method achieved the best imaging performance with the lowest recovery error (Table 1 and Table 2) compared to other methods.

There are some limitations that need to be addressed in future work. Firstly, the parameters for the T-FISTA and DC-FL methods, as well as the construction parameters of **W**_d_ and **W**_m_, were selected based on our previous study [24]. Automated parameter selection will be investigated. Secondly, for low photon counts, model-based frameworks, such as the Poisson distribution model [38], will be considered. Thirdly, as depth differences increase, such as at the 90° imaging view (the imaging setup of 0° view was shown in Figure 2b) in this study, the current depth compensation method fails to reveal targets that are far from the EMCCD. This issue will be addressed by incorporating additional priors. In addition, to achieve more accurate morphological reconstructions to obtain higher DICE values, more priors may be considered, such as group sparsity [39] or the luminescent intensity of adjacent voxels [40]. Overall, future work will focus on automated parameter selection, modeling low-flux statistics, and enhancing depth compensation to accommodate larger depth variations. Addressing these limitations will further enhance the proposed method’s utility for practical biomedical XLCT applications.

In conclusion, we propose a reconstruction method for single-view CB-XLCT. This method combines an automated restarting strategy with depth compensation. This approach enables high-quality imaging with high time resolutions as only a single view of data is required. Future work will focus on implementing the method for in vivo imaging, which could provide the rapid 3−D visualization of dynamic molecular processes.

## Figures and Tables

**Table 1:** Quantitative evaluation of simulations (EED = 0.1 cm).

	LE (mm)		DICE		SPI
	Tumor 1	Tumor 2	Tumor 1	Tumor 2	
T-FISTA	2.84	7.32	0.38	0.15	0.46
DC-FL	1.84	5.60	0.44	0.30	0.21
re-DC-FPGD	0.50	0.30	0.60	0.69	0.67

**Table 2:** Quantitative evaluation of the phantom experiments.

Method	LE (mm)		DICE		SPI
	Tube 1	Tube 2	Tube 1	Tube 2	
T-FISTA	1.95	4.98	0.47	0.01	0.12
DC-FL	2.06	4.87	0.32	0.06	0.16
re-DC-FPGD	0.20	0.83	0.59	0.71	0.99

## Data Availability

The data presented in this study are available on request from the corresponding author.
